# Peptide Mediated In Vivo Tumor Targeting of Nanoparticles through Optimization in Single and Multilayer In Vitro Cell Models

**DOI:** 10.3390/cancers10030084

**Published:** 2018-03-20

**Authors:** Celina Yang, Kyle Bromma, Devika Chithrani

**Affiliations:** 1 Department of Biomedical Physics, Ryerson University, Toronto, ON M5B 2K3, Canada; celina.yang@ryerson.ca; 2 Department of Physics and Astronomy, University of Victoria, Victoria, BC V8P 5C2, Canada; kbromma@uvic.ca

**Keywords:** gold nanoparticles (GNP), polyethylene glycol (PEG), multicellular layer (MCL)

## Abstract

Optimizing the interface between nanoparticles (NPs) and the biological environment at various levels should be considered for improving delivery of NPs to the target tumor area. For NPs to be successfully delivered to cancer cells, NPs needs to be functionalized for circulation through the blood vessels. In this study, accumulation of Polyethylene Glycol (PEG) functionalized gold nanoparticles (GNPs) was first tested using in vitro monolayer cells and multilayer cell models prior to in vivo models. A diameter of 10 nm sized GNP was selected for this study for sufficient penetration through tumor tissue. The surfaces of the GNPs were modified with PEG molecules, to improve circulation time by reducing non-specific uptake by the reticuloendothelial system (RES) in animal models, and with a peptide containing integrin binding domain, RGD (arginyl-glycyl-aspartic acid), to improve internalization at the cellular level. A 10–12% accumulation of the injected GNP dose within the tumor was observed in vivo and the GNPs remained within the tumor tissue up to 72 h. This study suggests an in vitro platform for optimizing the accumulation of NP complexes in cells and tissue structures before testing them in animal models. Higher accumulation within the tumor in vivo upon surface modification is a promising outcome for future applications where GNPs can be used for drug delivery and radiation therapy.

## 1. Introduction

Cancer is one of the leading causes of death worldwide. The incidence and death rates for several cancer types, including pancreatic cancer, are on an incline with a 5-year survival rate of less than 8% [1]. Radiation therapy and chemotherapy are the most common treatment modalities for cancer, along with surgery. The fundamental objective of all cancer therapeutics is to enhance tumor cell death, while minimizing normal tissue toxicity to improve the therapeutic index [2]. Despite successful clinical application of combined radiation therapy and chemotherapy, the major limitation of combining chemotherapy and radiation therapy is normal-tissue toxicity, since either modality can cause major normal tissue toxicity [2,3]. Due to the limitations of the current cancer treatment modalities, methods for improving the therapeutic results are continuously being researched. Gold nanoparticles (GNPs) are one of the materials that are used extensively in the field of nanomedicine and cancer research [4]. The enhanced tumor accumulation of biocompatible agents, such as GNPs, results in an improved therapeutic index [2]. Further improving bioavailability of GNPs through surface modification can potentially improve the therapeutic window of GNP mediated cancer therapy though co-delivery of chemotherapeutic agents and tumor-specific radiosensitization. Polyethylene glycol (PEG) is a commonly used molecule to decrease the nanoparticle NP surface exposure to proteins, such as opsonin [5]. Opsonin is a protein that binds to foreign microorganisms for enhanced phagocytosis and removal of foreign material from circulation for clearance. Protecting NP surfaces from binding of opsonin can improve blood circulation of the NPs [5,6]. Prolonged circulation of PEG coated particles promote tumor selectivity of NPs [7]. The limitations of modifying GNPs with PEG molecules is that accumulation on a cellular level is decreased because the PEG moiety hinders binding of PEGylated NPs to tumor cell receptors [5,6,7].

**Figure 1:**
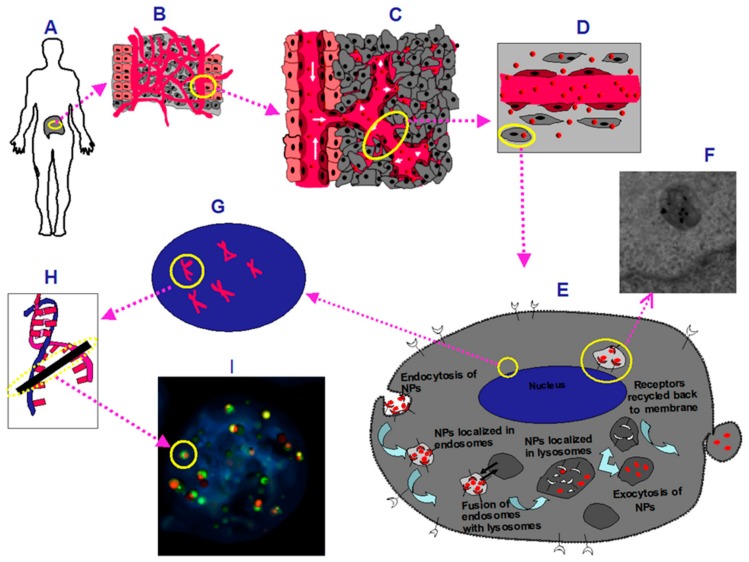
A schematic of gold nanoparticle (GNP) accumulation from vasculature to cell level (**A**) A diagram representing a pancreatic tumor; (**B**) Tumor microenvironment; (**C**) Abnormal tumor vasculature; (**D**) GNPs (represented as red spheres) leaking through tumor vessels; (**E**) A diagram that represents one of the GNP cell uptake pathways.; (**F**) Transmission Electron Microscopy (TEM) images of GNPs in a vesicle close to the nucleus of a cell; (**G**) Enlarged schematic of the nucleus with DNA represented as double helices; (**H**) A double strand break in the DNA is represented. One of the major targets and mechanisms of radiation and many chemotherapeutic agents is to kill cancer cells through DNA breaks; (**I**) Markers that probe for DNA double strand breaks (DSB).

NP surface modifications can be further achieved with antibodies that have been used to target the NPs to specific cancer cells. For example, epidermal growth factor receptor (EGFR) has been used to target GNPs into pancreatic cancer cell lines in both in vitro and in xenograft mouse models [8]. However, antibodies can be relatively large and limit the number of bindings onto each NP [8]. The use of peptides, which are smaller than antibodies, as targeting ligands provides room for multiple binding sites for other different cargo which can be used for facilitating novel approaches for cancer therapy [9]. One of the peptides that has been used in targeting pancreatic tumors contains a tripeptide sequence, arginyl-glycyl-aspartic acid (RGD) (Arg-Gly-Asp). The RGD tripeptide sequence is found in proteins such as fibronectin, citronectin, and type I Collagen [10,11]. These three amino acids form the core structure recognized by cell surface receptors and can improve the intracellular retention of the NPs [11,12]. Improved tumor targeting has been observed in studies using RGD-modified drug constructs because the integrin receptors are overexpressed on the surface of most types of cancer cells and RGD modified particles can enter the cytoplasm through receptor-mediated endocytosis [13,14,15,16,17,18]. RGD peptides have been used to target GNPs to oral cancer cells in vitro [19], and contrast agents have been functionalized with RGD peptides for imaging tumor vasculature [20]. In this study, RGD containing integrin binding domain is conjugated on the GNP surface to target the tumor vasculature and the extracellular matrix of a pancreatic tumor. Our previous studies showed that GNP uptake and transport varies as a function of size and surface properties at monolayer and multilayer tissue-like levels [21,22]. Hence, we optimized our NP systems both at the monolayer and multilayer tissue-like levels before testing them in vivo. This is the first time that a NP platform has been tested both at monolayer and multilayer levels before testing in vivo. As illustrated in schematic Figure 1, successful delivery of NPs into a tumor depends on the efficiency of crossing a few boundaries: (a) Nanoparticles should be properly functionalized to avoid macrophage uptake and enhance the circulation lifetime; (b) Once they reach the tumor blood vessels, they can make use of the leakiness of the vasculature to enter tumor tissue; penetration of NPs within the extra-cellular matrix (ECM) is also an important factor; (c) finally, NPs should be able to enter individual tumor cells efficiently to deliver the therapeutic load. In most cancer therapeutic techniques, the goal is to damage DNA of the tumor cells. Gold nanoparticles can play a key role in cancer therapeutics since GNPs can be used to enhance the radiation dose in radiation therapy and controlled delivery of anticancer drugs in chemotherapy. In this study, we optimized the GNP uptake at both monolayer and tissue level using in vitro models before testing them in animal models. This type of approach could be used to bridge the gap between the in vitro models and in vivo studies.

## 2. Results

### 2.1. Characterization of Gold Nanoparticles Functionalized with Peptide and PEG Molecules

**Figure 2:**
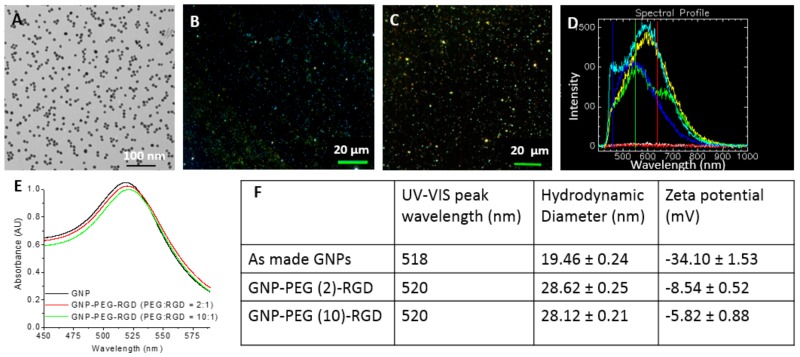
Characterization of GNPs used in this study: (**A**) Transmission Electron Microscopy (TEM) of GNPs used in this study. Scale bar = 100 nm; (**B**) Dark field image of GNPs. Scale bar = 20 µm; (**C**) Hyperspectral image of GNPs; (**D**) A few spectra of the GNPs from (**C**) and background spectra; (**E**) Ultraviolet-Visible (UV-VIS) spectra of GNP constructs; (**F**) Peak wavelength, hydrodynamic diameter, and zeta potential of GNP constructs used in this study.

The shape and size of GNPs used for this study was determined with Transmission Electron Microscopy (TEM) and Ultraviolet-Visible (UV-Vis) spectroscopy as shown in Figure 2A,E. The approximate core diameter of these GNPs was 9.8 ± 0.4 nm obtained from the TEM image (Figure 2A). A dark field and Hyper Spectral Imaging (HSI) images of 10-nm GNPs are shown in Figure 2B,C, respectively. The bright dot-like structures are GNPs. Each pixel of the GNP image in Figure 2C consists of spectral information and some reflectance spectra collected from those bright spots are shown in Figure 2D. Spectra collected from GNPs had high intensity while the ones collected from the background were flat. The UV-Vis spectra had a peak at Surface Plasmon Resonance (SPR) wavelength of approximately 518 nm (Figure 2E and 5). The peak wavelength of 518 nm for 10 nm sized GNPs is within agreement with the wavelength-NP size correlation [23,24]. UV-Vis measurements can also be used to evaluate the functionalization or aggregation of GNPs. The shape of the UV-Vis spectra shown in Figure 2E did not significantly differ for unmodified GNPs, and GNPs modified with both PEG and RGD peptide. The RGD peptide and PEG molecules used for conjugation were relatively small (molecular weight was 1760 and 2000 Da, respectively). This resulted in a slight increase in the hydrodynamic diameter and a 2 nm shift in the UV peak wavelength. Functionalization of GNPs with PEG and RGD peptide molecules were done at ratios of 2:1 and 10:1, respectively. These two complexes are labelled as GNP-PEG-RGD (2:1) and GNP-PEG-RGD (10:1). There was no significant broadening of the UV spectra up to 46 h post-formulation, and this indicates the NP complexes were stable (Figure 2E). However, there was a decrease in the total negative charge (zeta potential) of the GNPs once they were functionalized with PEG and RGD (Figure 2F). The decrease in total negative charge is predicted to be due to the displacement of negatively charged citrate capping ligands with neutral PEG and positively charged RGD peptide molecules [25]. 

### 2.2. Accumulation of Gold Nanoparticles at the Monolayer Level

**Figure 3:**
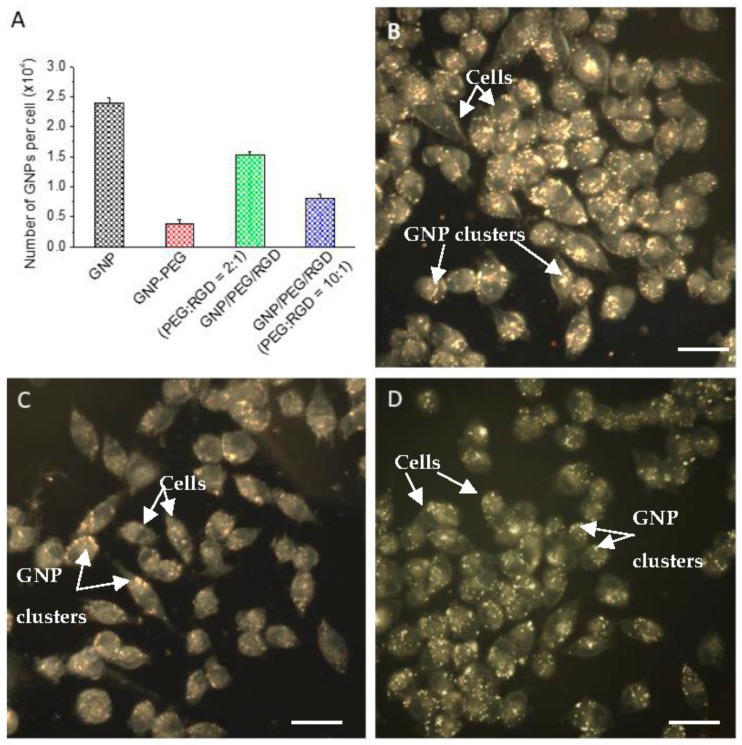
(**A**) Accumulation of GNP constructs in monolayer cells; (**B**) Dark field image of cells incubated with unmodified GNPs; (**C**) Dark field image of cells incubated with GNP-PEG; (**D**) Dark field image of cells incubated with GNP-PEG-RGD (2:1). Scale bar = 10 µm. PEG = polyethylene glycol; RGD = arginyl-glycyl-aspartic acid.

As shown in Figure 1E, an endosomal compartment is formed as the particles are internalized. Although several different uptake pathways have been suggested for GNP cell internalization, the major internalization pathway of unmodified GNPs in cells is reported to be an energy dependent process [22,26,27,28,29], as the uptake of GNPs decreases in low temperature (4 °C) and other ATP-depleted environments, such as in cells pre-treated with sodium azide (NaN_3_) [27,30,31,32,33]. It appears that particles are subsequently recycled back to the plasma membrane or progress to lysosomes for degradation [34]. Figure 3A illustrates the cellular accumulation of unmodified (citrate-capped) GNP, GNP-PEG, and GNP-PEG-RGD after 16-h incubation in pancreatic cancer (MIA-PaCa-2) cells. The accumulated number of unmodified GNP, GNP-PEG, GNP-PEG-RGD (2:1) and GNP-PEG-RGD (10:1) were 23,800 ± 800, 3600 ± 500, 15,250 ± 600, and 8000 ± 700, respectively. The surfaces of GNPs were modified with PEG molecules to reduce the RES uptake by macrophages during in vivo experiments. However, PEG molecules on the GNP surface reduced the NP uptake significantly. This trend was also observed by comparing the dark-field optical images in Figure 3B,C. The bright structures are GNP constructs and it can be observed that the bright structured in Figure 3C are reduced in Figure 3B. Conjugation of RGD peptides onto the GNP surface resulted in a significant increase in the NP accumulation, as shown in Figure 3A. This increase in accumulation upon RGD peptide conjugation was also observed by comparing the dark field images in Figure 3C,D. There was a 50% reduction in the NP uptake when PEG:RGD ratio was increased to 10:1 from 2:1. This shows that the RGD peptides influences NP accumulation.

### 2.3. Accumulation of Gold Nanoparticles in Multilayer the Model

**Figure 4:**
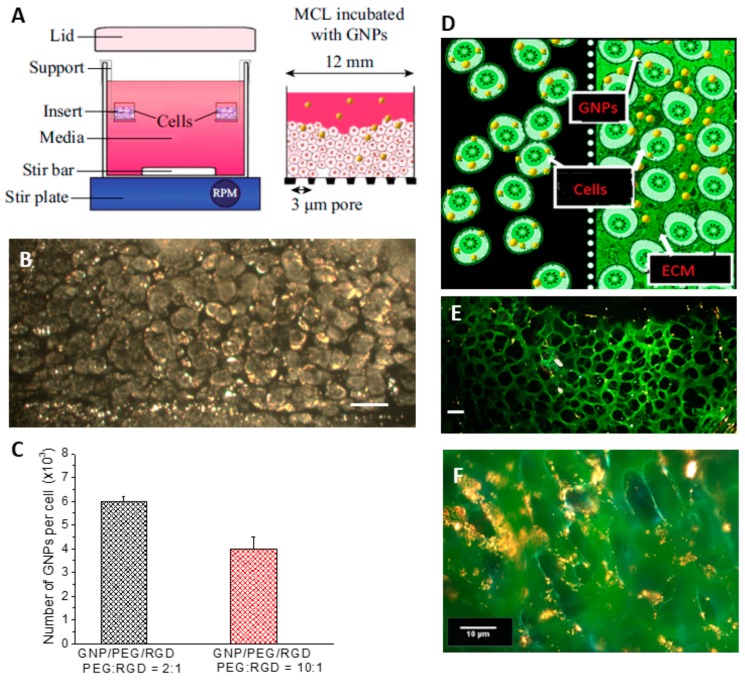
(**A**) Setup of multilayer model (MCL); (**B**) Dark field image of MCL. Scale bar = 10 µm; (**C**) Accumulation of GNP constructs in MCL; (**D**) Illustration of the comparison of GNP accumulation in monolayer (left) and MCL (right). ECM = extracellular matrix; (**E**) MCL stained with eosin. Scale bar = 10 µm; (**F**) MCL stained with eosin. Scale bar = 10 µm.

Figure 4A is a schematic diagram of the multilayer model experimental setup and the insert used for the multicellular layer (MCL) growth. As shown in Figure 4A, tissue culture inserts were held suspended while the media was constantly stirred (left). After the MCL growth, GNPs were introduced into the media to investigate the GNP transport through the layers (right). Tissue-like MCLs are designed to mimic the environment of solid tumor tissue and are used as a model to observe the GNP transport and accumulation in tumor tissue. The dark field image of an unstained multilayer cross section is shown in Figure 4B. The bright structures are gold nanoparticle clusters. The quantification data in Figure 4C indicate that there is a decrease in the accumulation of the two GNP complexes per cell in the 3D MCL by 60% (PEG:RGD ratio of 2:1) and 50% (PEG:RGD ratio of 10:1) compared to monolayer cell cultures (Figure 3A), respectively. The decrease is predicted to be due to the presence of the Extra Cellular Matrix (ECM; marked in green) heavily present in between the layers of the multilayer model (right) as opposed to the monolayer cell cultures (left), as shown in the schematic in Figure 4D. GNPs incubated in the MCL culture have to penetrate through the ECM before accessing the cells (Figure 4D right), while the lack of ECM in monolayer cells allow more GNPs to accumulate in cells (Figure 4D left). To visualize the ECM, the tissue cross sections were stained with eosin. Eosin is a florescent dye used for staining proteins in the cytoplasm of the cells and collagen in the ECM. Figure 4E is a dark field image of a thin tissue cross-section stained with eosin while Figure 4F represents a thicker tissue cross-section. However, NPs (appear as a yellow color) were still transported through the ECM, as seen in Figure 4E,F.

**Figure 5:**
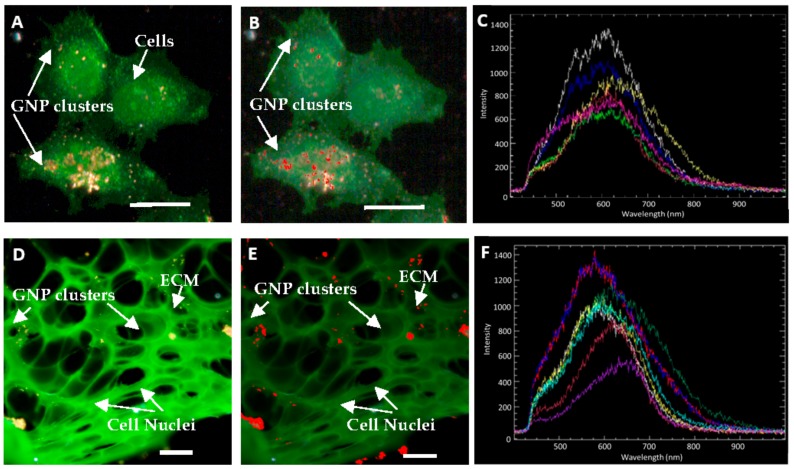
Comparison of monolayer and multilayer cell accumulation of GNPs (**A**) Dark field image of GNP accumulation in monolayer cells; (**B**) Hyperspectral image of GNP accumulation in monolayer cells. Pixels that are identified as GNPs are marked in red; (**C**) A few spectra collected from GNP clusters in Figure **B**; (**D**) Dark field image of GNP accumulation in multilayer cell model.; (**E**) Hyperspectral image of GNP accumulation in a multilayer cell model. Pixels that are identified as GNPs are marked in red; (**F**) A few spectra of GNP clusters collected from Figure (**E**). Scale bars = 10 µm.

The presence of GNP clusters in monolayer and multilayer structures, as shown in Figure 5, was verified using hyperspectral imaging technology. Figure 5A is a dark field image of a few cells in a monolayer cell culture while Figure 5B is an HSI image of the cells shown in Figure 5A. Marked in red on Figure 5B are GNPs within cells mapped using the reference GNP spectrum (marked in white color) shown in Figure 5C. A few reflectance spectra collected from GNP clusters within cells in Figure 5B are shown in Figure 5C. Similarly, Figure 5D–F represent a dark field image, an HSI image, and a few reflectance spectra collected from GNP clusters in the multilayer cell model.

### 2.4. In Vivo Accumulation and Pharmacokinetics of GNPs in a Pancreatic Cancer Model

**Figure 6:**
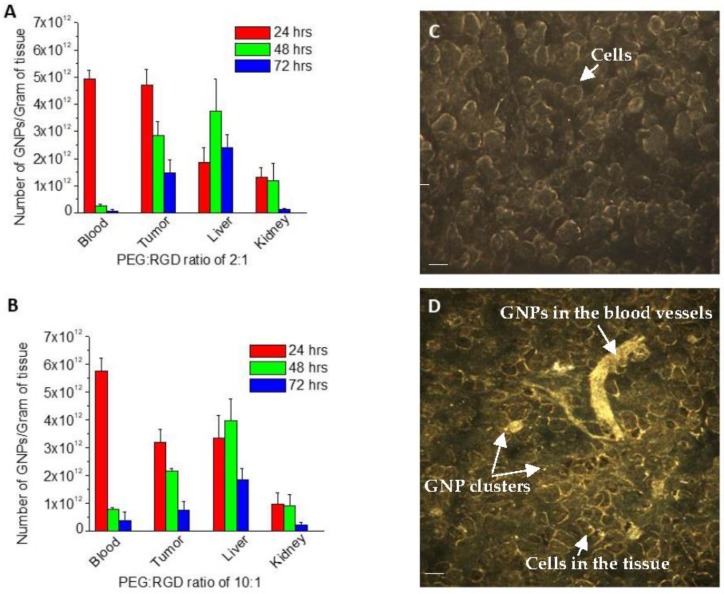
(**A**) Accumulation of GNP-PEG-RGD (2:1) in various organs of severe combined immunodeficiency (SCID) mice; (**B**) Accumulation of GNP-PEG-RGD (2:1) in various organs of SCID mice; (**C**) GNP accumulation in tumor immediately post-injection. Scale bar = 10 µm; (**D**) GNP accumulation in tumor 24 h post injection. Scale bar = 10 µm.

Figure 6A,B illustrates the pharmacokinetic profiles of GNPs at both the tumor and plasma level in the in vivo pancreatic cancer model. We used GNP-PEG-RGD with 2:1 and 10:1 ratios. The highest accumulation was observed 24 h after injection of the GNPs with a subsequent decrease post 48 h and 72 h. Tumor accumulation was observed at a maximal level of 4.74 × 10^12^ GNPs/g (≅12.8% injected dose) and 3.19 × 10^12^ GNPs/g (≅8.6% injected dose), for the 2:1 and 10:1 formulations, respectively. Figure 6C,D shows the accumulation of GNPs in a section of tumor through dark field images immediately after and 24 h after injection. The maximum serum concentration of GNPs post-injection (C_max_) for the preceding groups are 1.53 × 10^10^ and 1.80 × 10^10^ GNPs/µL and the time of peak GNP serum concentration (t_max_) is 24 h for both formulations. The biological half-life following first order kinetics was calculated to be 9.08 h and 8.34 h for the 2:1 and 10:1 ratios, respectively. The clearance of the two formulations are 7.63 µL/h and 8.31 µL/h, respectively. The area under the plasma-GNP concentration-time curve (AUC) values are 1.95 × 10^12^ (2:1) and 1.81 × 10^12^ GNPs µL/h (10:1). The 2:1 PEG-RGD formulation is experimentally shown to have an improved tumor uptake with the 10:1 demonstrating marginally ameliorated pharmacokinetics. The half-life in both groups is sufficiently low, indicating fast pharmacokinetics and signifying that any toxicity observed in other organ systems will be minimal. Albeit, the tumor uptake remains relatively high at 48 h post injection in both groups, elucidating that a significant amount of the modified GNPs remain for a prolonged period. Both formulations have a maximal concentration at 24 h, with the clearance rate of the 10:1 PEG-RGD being slightly higher, confirmed by the lower half-life.

**Figure 7:**
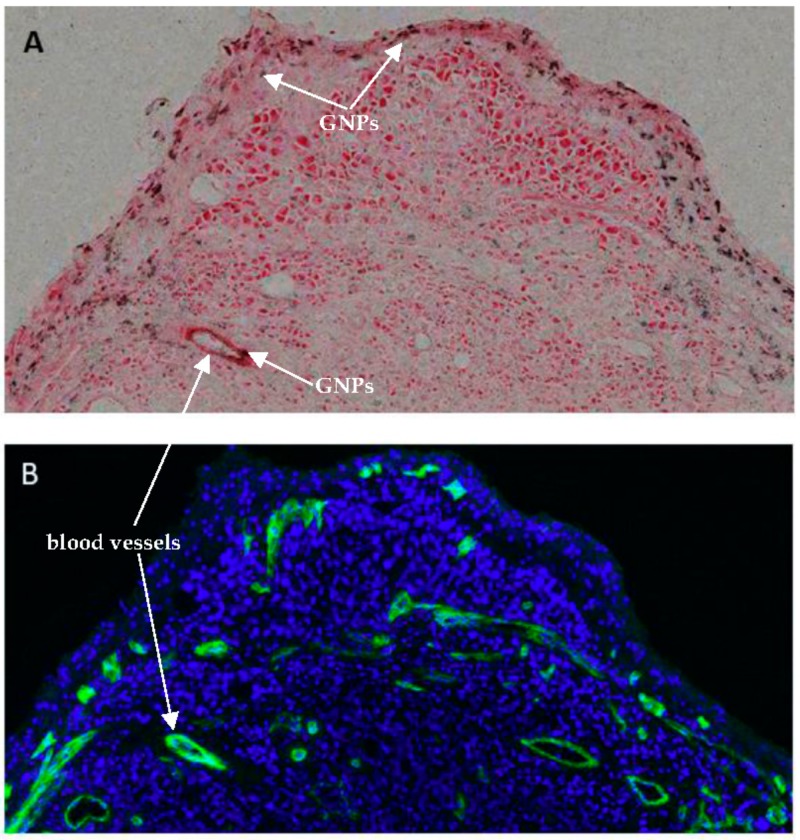
GNP accumulation in tumor (**A**) silver stained GNPs (shown in black) accumulated in eosin stained section of tumor; (**B**) section of tumor with cell nucleus stained with DAPI (4′,6-Diamidino-2-phenylindole dihydrochloride; shown in blue) and blood vessels marked with CD31 (shown in green).

Figure 7 further illustrates GNP accumulation in a tumor with respect to blood vessels. Figure 7A is a section of tumor stained with eosin (shown in red) with silver stained GNP constructs shown in black. The deposition of silver on the gold nanoparticle surface selectively alters the appearance of GNPs and allows detection using a standard light microscope. Figure 7B is the same tumor section, where the cell nuclei are shown in blue, and the blood vessels are stained with a CD31 marker shown in green. Most GNP accumulation occurred near the blood vessels, and there were more blood vessels observed in the tumor periphery than in the center of the tumor.

**Figure 8:**
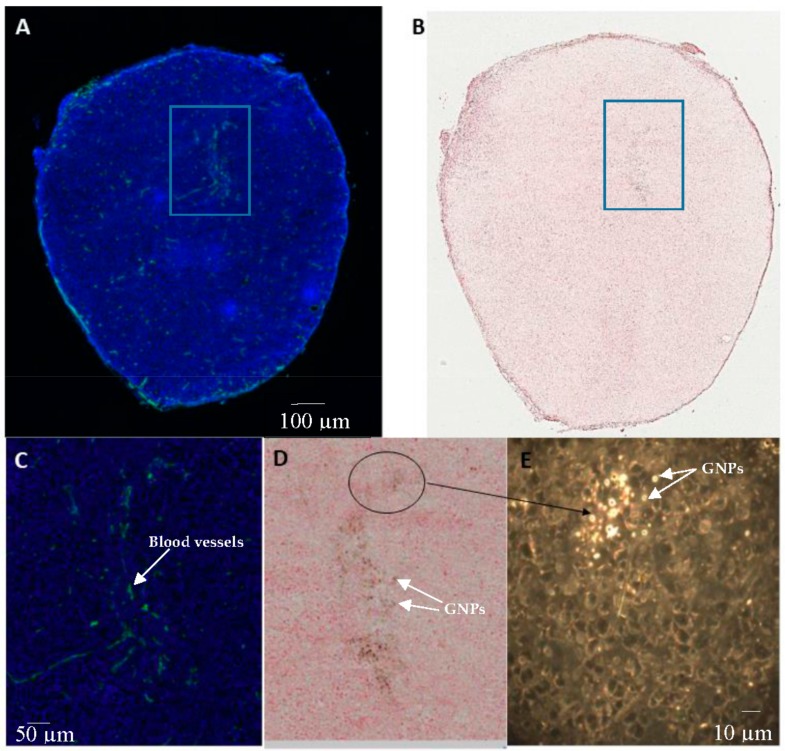
GNP accumulation in a tumor: (**A**) section of tumor with cell nucleus stained with DAPI (blue) and blood vessels marked with CD31 (shown in green); (**B**) silver stained GNPs (shown in black) accumulated in an eosin stained section of tumor; (**C**) magnified image of (**A**); (**D**) magnified image of (**B**); (**E**) dark field image corresponding to (**D**).

Although more blood vessels were observed in the tumor periphery than the center of the tumor, blood vessels were still found within the tumor center. Figure 8 further illustrated GNP accumulation in the tumor with respect to blood vessels. Figure 8A,C are of a section of tumor, where the cell nuclei is shown in blue and the blood vessels are stained with a CD31 marker shown in green. Figure 8B,D are of the same tumor section stained with eosin, and GNP constructs are shown in black through silver staining. GNP accumulation within the tumor was heterogeneous with more accumulation close to the blood vessels as shown by the overlap of blood vessels in green (Figure 8A,C) with the GNP shown in black (Figure 8B,D). The corresponding dark field image of Figure 8C,D is shown in E, where the GNPs are shown as bright structures. It can be confirmed from this dark field image that GNP accumulation within the tumor is heterogeneous. However, GNPs were still observed throughout the tumor.

## 3. Discussion

The surfaces of GNPs in this study were modified with PEG molecules to reduce the reticuloendothelial system (RES) uptake by macrophages during in vivo experiments. However, PEG molecules on the GNP surface reduce the NP uptake significantly, which was also observed in this study as shown in Figure 3B–D. The limitations of modifying GNPs with PEG molecules is that the accumulation at the cellular level is decreased because the PEG moiety hinders binding of PEGylated NPs to tumor cell receptors [5,6,7]. Based on previous findings, cellular uptake of PEG coated NPs was improved by introducing a peptide containing integrin binding domain, RGD [5,6,25]. For example, Cruje et al. also observed the 14 nm RGD-PEG modified GNP constructs had approximately a 29% accumulation compared to that of unmodified citrated stabilized GNPs in MDA-MB-231 breast cancer cells [5]. The RGD peptide on the NP surface can act as a driving force during the internalization process since the RGD domain is recognized by cell surface receptors [11]. This RGD peptide domain is known to target integrin proteins, which are overexpressed on the surface of most types of cancer cells [17,18]. Conjugation of RGD peptides onto the GNP surface resulted in a significant increase in the GNP accumulation in this study, as shown in Figure 3A. This increase in accumulation upon RGD peptide conjugation was also observed from the dark field images in Figure 3C,D. There was a 50% reduction in the GNP uptake when the PEG:RGD ratio was increased to 10:1 from 2:1 (Figure 3A). This shows that the RGD peptides influence GNP uptake. While it was expected that a higher cell accumulation of the GNP constructs would be observable with more RGD peptides, there is a limit to varying the PEG:RGD ratio. Since PEG acts as a stabilizer of the GNP constructs, reducing the PEG ratio can cause aggregation. Schulz et al. have observed that decreasing the PEG molecules compared to neural cell adhesion molecule L1 on GNP surfaces is correlated with aggregation of the complexes [35]. Although the mechanism is unclear, aggregation of particles have been shown to influence cellular uptake and interaction [36]. The PEG and RGD modified GNPs remain stable in buffer solution, and the UV-VIS spectra, Dynamic Light Scattering (DLS) measurements, and zeta potential are shown in the Supplementary Materials.

It is generally recognized that in vitro results cannot be extrapolated directly to in vivo settings [37]. Several extra factors must be considered to extend in vitro work to in vivo studies. Accumulation of the NPs into the cells becomes a more complicated process as NPs must pass more barriers before entering the cells [38]. As a result, the outcome of NP-based research is sometimes overstated, and in vivo trials fall short of expectations [39,40]. After observing the accumulation of the GNP constructs at the monolayer level, the next goal was to test the NP system in a 3-Dimensional in vitro tissue model before using them in animal experiments. Multicellular spheroid structures are commonly used for 3D cell cultures, however, the spheroid geometry is the reverse of the general in vivo physiology and makes it difficult to assess the penetration of drugs in a solid tumor. While in the spheroid geometry, drugs and agents pass radially inwards from the edges of the spheroid, the multilayer model in this study provides a top-down or side-to-side geometry and better mimics the distribution of agents through tissue once they leave the blood vessel [41,42,43]. Hence, the multilayer model used in this study is ideal for examining the penetration and transport of agents through such tissue. Figure 4B is a dark field image of an unstained tissue cross-section and the penetration of the NPs can be seen through the tissue.

As illustrated in Figure 4 and Figure 5, GNPs still penetrated through the extracellular matrix (ECM). The ECM is an essential component of tissues that consists of collagen and elastin fibers immersed in visoelastic gel that is composed mainly of hyaluronan and proteoglycan [44]. This matrix of collagen networks prevents penetration of macromolecules and the delivery of therapeutic agents to cancer cells [45,46]. Serum proteins that adsorb on the surface of NPs can be cross-linked into tissue through any available amine groups found at the N-terminus in lysine residues [47]. While this cross-linking helps NPs stay in the tissue, it prevents the further penetration of NPs. A tumor with a well-developed collagen network can be considered to be physically resistant to macromolecule-based therapies [45]. Our group previously observed that the GNPs penetrated deeper through the breast cancer tissue of MDA-MB-231 cells, which has less organized ECM, than through breast cancer tissue of MCF-7 cells [21]. This study showed that a better developed ECM prevents penetration of nanoparticles. The development of the collagen network varies from cell line to cell line, but degeneration of the collagen network and an abnormal ECM organization can be common features of tumors [45]. The pancreatic cancer ECM is composed of collagens, non-collagen glycoproteins, glycosamminoglycans, growth factors, and proteoglycans as well as modulators of the cell matrix interaction [48]. It has also been reported that the ECM of pancreatic tumor tissue is non-uniform using 3D tissue cultures [49,50]. The characteristic of tumors having abnormal ECM can be advantageous in having NPs accumulated more in the tumor tissues than in the normal tissues. For tumors with a relatively well-developed collagen network, treatments that reverse or inhibit collagen production and assembly can be performed prior to macromolecule-based therapies [45]. Although there was a decrease in accumulation of the GNP constructs incubated in multicellular models, the GNPs were still transported through the ECM as shown in Figure 4E,F. The bright structures are GNP constructs and can be observed throughout the multicellular layers. The comparison of GNP accumulation in monolayer cells and multilayer cells is illustrated in Figure 4D.

Based on in vivo accumulation and pharmacokinetics of GNPs in pancreatic cancer model studies, the accumulation of the GNP constructs in the tumor was highest at 24 h post injection, which decreased subsequently as shown in Figure 6A,B. The clearance from the tumor was lower than the clearance from the blood, which indicates that the GNP retention in the tumor is longer than GNP clearance from circulation. The sufficiently low half-life of the GNP constructs from the first pharmacokinetics suggest that toxicity to other organ systems will be minimal with this initial injection dose. A comprehensive review by Wilhelm et al. analyzed the NP tumor delivery efficiency from current NP delivery publications [51]. The median and mean of gold material tumor uptake in (% Injected Dose/g) were 3.5 and 6.1, respectively [51]. The discrepancy between the median and mean indicate a high variability of data sets [51]. The large variability between studies indicates that accumulation of GNPs is dependent on multiple parameters, including the physicochemical properties of the GNPs (size, shape, surface chemistry), presence of targeting molecules, and the tumor environment (size and type of tumor, properties of the tumor blood vessels involved, and properties of ECM involved, etc.). The varied parameters used across different studies make it challenging to compare results in a meaningful way and to identify the optimal parameters.

It can be observed from Figure 7 and Figure 8 that GNP accumulation in tumors is heterogenous, where more GNPs are observed in and near blood vessels. CD31 is an endothelial maker often used in determining tumor vascular density. The tumor vessels stained with CD31 are shown in green (Figure 7B and Figure 8A,C). The GNPs are shown in black in Figure 7A and Figure 8B,D. It was observed that the GNPs mostly overlap with the vessels. From Figure 7, GNP accumulation was observed to be highest in the tumor periphery, and the periphery of the tumor was where the vessels were most abundant. Tumor blood vessels are generally more abundant at the periphery than in central regions [52]. However, vessels were still found in the center of the tumor, as shown in Figure 8, therefore, although heterogenous, GNPs were found throughout the tumor.

Modifying the GNP constructs with PEG reduced the accumulation at monolayer cell models. These results were expected as it has also been shown from previous studies that GNP modification with PEG decreases cell accumulation [5,6,7]. Despite the low accumulation at cellular levels, PEG is commonly used to functionalize GNP surfaces as it can decrease the NP surface exposure to proteins, such as opsonin, and improve blood circulation in animal studies [5,6]. Although varied parameters used across different studies make it challenging to compare results, the tumor accumulation of GNP-PEG-RGD was comparable to a study that used similar sized PEG functionalized GNPs. Also, the GNP retention was observed to be longer than GNP clearance from circulation, which suggests GNPs can be retained in the tumor for the duration of treatments to be used with other therapeutic modalities. The results from this study have shown that the GNP retention in tumors is significant after 24, 48, and 72 h post injection, as compared to the amount that is cleared from the circulation. The results from this study indicate that GNP structures developed in monolayer studies can be used in multilayer and in vivo studies. Further modifications can be done in each translation step to optimize the use of the constructs.

## 4. Materials and Methods

### 4.1. Synthesis and Characterization of NPs

Gold nanoparticles were synthesized using the citrate reduction method [53]. First, 300 μL of 1% chloroauric acid (HAuCl_4_·3H_2_O) (Sigma-Aldrich, Oakville, ON, Canada) was added to 30 mL of double–distilled water and heated on a hot plate while stirring. Once it reached the boiling point, 1 mL of 1% sodium citrate tribasic dehydrate (HOC(COONa)(CH_2_COONa)_2_·2H_2_O) (Sigma-Aldrich) was added. After the color of the solution changed from dark blue to bright red, the solution was left to boil for another 5 min while being stirred. Finally, the GNP solution was brought to room temperature while being stirred.

The GNPs were characterized by Transmission Electron Microscopy (TEM) (H7000; Hitachi Corp., Tokyo, Japan), UV-spectroscopy (Lambda 40; PerkinElmer, Waltham, MA, USA), Hyper Spectral Imaging (HSI), and by Dynamic Light Scattering (DLS) using a 90 Plus Particle Sizer Analyzer (Brookhaven Instruments Corp., New York, NY, USA) to determine the size of the NPs.

### 4.2. Conjugation of Peptides and PEG onto GNPs

A 0.1% PEG solution was prepared with thiol-terminated polyethylene glycol with a molecular weight of 2 kDa. The solution was added to GNP solutions to achieve a grafting density of 2 PEG molecules per nm^2^. For 10 nm GNPs, approximately 630 or 3150 PEG molecules were added. A solution of peptide sequence, CKKKKKKGG**RGD**MFG was mixed with a solution of the PEG molecules at PEG:RGD = 2:1 or 10:1 ratio. The PEG and RGD is conjugated onto the GNP surface through a gold-thiol bond. To confirm PEGylation of GNPs, DLS measurements were conducted. This was followed by UV-visible spectrophotometry to confirm the stability as shown in the Supplementary Figure S1.

### 4.3. Cellular Accumulation Studies

Human pancreas cancer cells (MIA-PaCa-2, ATCC#: CRL-1420™) were cultured in Dulbecco’s Modified Eagle’s Medium (DMEM) with 10% Fetal Bovine Serum and grown to confluent so that three wells of a 6-well tissue culture dish were incubated with the same NP type. For optical imaging purposes, MDA-MB-231 cells were placed on glass coverslips and grown to 60% confluent. Cell cultures were incubated with 5 × 10^10^ GNPs per dish for 14 h. Following incubation, all cell cultures were washed with Phosphate-Buffered Saline (PBS) three times. Those without coverslips were trypsinized and processed for quantification studies. Those with coverslips were rinsed twice with PBS, followed by fixation with 4% paraformaldehyde in PBS for 10 min at room temperature, then rehydrated in PBS. Coverslips were mounted onto glass slides and were dried overnight and kept at 4 °C prior to being imaged.

### 4.4. Growth of Multi-Cellular Layers (MCLs)

The growth of the MCLs began with the growth of monolayer cells (MIA-PaCa-2, ATCC#: CRL-1420™) in a 5% CO_2_ environment at 37 °C. After reaching confluence, these cells were trypsinized, centrifuged, suspended in media, and counted. Approximately 150,000–200,000 cells were seeded onto a microporous membrane insert (Millicell, Bedford, MA, USA). After allowing the cells to attach for 2 to 4 h, the inserts were washed with phosphate buffered saline (PBS) and then suspended in stirred media to grow. With pore sizes of 3 µm, the inserts allowed for the passage of stirred media through the base of the insert. Cells were grown on the MCL insert in Dulbecco′s Modified Eagle′s Medium (Life Technologies Inc., Burlington, ON, Canada) with 10% Fetal Bovine Serum (Sigma-Aldrich, Oakville, ON, Canada). The ECM within the tissue was stained with eosin for visualization (Figure 2C). The thickness of the tissue was controlled by the growth period. MCL incubation with NPs was done by hanging the MCLs in multi-well plates followed by filling the top of the inserts with the GNP and media mixture. A supply of fresh media was placed below the MCL to allow for GNPs that had penetrated the entire MCL structure to diffuse. The setup for growing MCLs are shown in Figure 4A.

### 4.5. Pancreatic Xenograft Model

Human pancreas cancer cell line (MIA-PaCa-2, ATCC#: CRL-1420™) was cultured in Dulbecco DMEM-Dulbecco’s Modified Eagle Medium (Life Technologies) together with 10% Fetal Bovine Serum (FBS), 100 units/mL penicillin G, and 100 μg/mL streptomycin (Hyclone). Cells were maintained at 37 °C in a humidified atmosphere containing 5% CO_2_. To derive subcutaneous (s.c.) xenografts, 6–8-week old female severe combined immunodeficiency (SCID) mice were injected with 1.5 × 10^6^ cells in the lower left dorsal flank. Injection of gold nanoparticles in the tail vein was started when xenografts reached a volume of ~250 mm^3^. Mice were randomly divided into groups for all studies and subsequently sacrificed at intervals of 24 h, 48 h, and 72 h. Tumor length and width measurements were converted into tumor volume using (L × W^2^/2) where L and W are the larger and smaller diameters, respectively; tumors were measured every 2 days with calipers. Animals were monitored for any signs of physical toxicity over the duration of each study. Experiments were conducted in accordance with the University Health Network (UHN) Animal Care Committee guidelines.

### 4.6. In Vivo Comprehensive Acute and Physical Toxicity Assay

SCID mice were injected with gold nanoparticle formulations and sacrificed 24 h after injection, and blood was collected from the tail vein; if this was not technically possible, the saphenous vein or direct terminal cardiac puncture was used as an appropriate substitute. Samples were then centrifuged at 14,000 rpm and serum was removed from the mixture and assessed for hepatotoxicity, nephrotoxicity, and electrolytes using an Autoanalyzer (Applied Biosystems, Burlington, ON, Canada). Mice were observed every 2 days for signs of general toxicity, which include, but are not limited to: body weight changes, dull sunken eyes, interrupted breathing, and lethargy. The results of the toxicity assay are shown in the Supplementary Table S2.

### 4.7. In Vivo Biodiversity Assay

SCID mice were sacrificed at pre-determined time-points and tumor and organs (liver, kidney, pancreas, spleen) were surgically removed and analyzed ex vivo. Subsequently, organs were homogenized using a mechanical homogenizer. All surgical and organ removal techniques were conducted in accordance with UHN Small Animal Surgery Guidelines.

### 4.8. Immunohistochemistry

All serial sections (50 µm separation between sections) were cut from frozen block tumor tissue. Sections were stained for CA9 (Rabbit Monoclonal, Thermo Scientific, Burlington, ON, Canada) and CD31 (provided by Dr. Cameron Koch, University of Pennsylvania). Secondary antibodies were used alone to control for nonspecific background. Sections were counterstained with 1 µg/mL DAPI (4′,6-Diamidino-2-phenylindole dihycrochloride) to outline the nuclear area. Images were scanned on the TS4000 (Huron Technologies, Waterloo, ON, Canada) at 0.5 µm/pixel. Regions of tumor, necrosis, stroma, and folds were specified, creating a training rule-set for tissue recognition using Tissue Studio (Definiens, Munich, Germany). Cellular analyses included nucleus identification and separation, objects <10 µm^2^ being excluded.

### 4.9. Quantification of GNPs

GNP accumulation in cells or organs was quantified using Inductively Coupled Plasma Atomic Emission Spectroscopy (ICP-AES). Following 16 h of incubation with GNPs, the cells were washed three times with PBS and the cells were suspended from the monolayer cultures with 0.25% trypsin-EDTA (Ethylenediaminetetraacetic acid) (Gibco, Burlington, ON, Canada) for quantification of GNPs present per cell. Cells were counted with either a hemocytometer (Hausser Scientific, Horsham, UK) or a Vi-CELL XR automated cell counter (Beckman Coulter, Brea, CA, USA). The surgically removed organs were weighed and homogenized. Then, the samples were treated with aqua regia (mixture of 37% hydrochloric acid (HCl) (Sigma-Aldrich) and 70% nitric acid (HNO_3_) (Caledon Laboratories Ltd., Georgetown, ON, Canada) at a ratio of 3:1 in a silica oil bath. The samples were diluted and concentrations of gold (Au) atoms were measured (mg/L) with the Optima 7300 DV ICP AES (Perkin Elmer, Waltham, MA, USA). The resulting gold atom counts were converted to GNPs per cell or per organ weight.

### 4.10. Hyperspectral Imaging

The CytoViva (CytoViva Inc., Auburn, AL, USA) technology in combination with dark field microscopy was used to image GNP distribution within cells. The illumination of the microscope system utilized oblique angle illumination to create high resolution dark field images. GNPs appear bright due to their high scattering cross-sections. This imaging technique does not require optical labeling with fluorophores on GNPs for detection, and it is also possible to extract spectral information from each pixel for verification purposes [12,21]. This imaging technique was used to image GNPs localized in cells, 3D tissue models, and tumor tissue cross-sections in animal models used in this study.

## 5. Conclusions

This study shows that optimizing of NP complexes both at the monolayer and tissue-like multilayer levels before testing them in animal models has many advantages. These multifaceted in vitro platforms can be further improved in the future to mimic a tumor microenvironment better. We believe that such improved in vitro platforms can accelerate successful implementation of NP complexes in biomedical applications through better understanding of the complex tumor microenvironment.

## Supplementary Materials

The supplementary materials are available online at http://www.mdpi.com/2072-6694/10/3/84/s1.
