# Supplementary material for: Peptide Mediated In Vivo Tumor Targeting of Nanoparticles through Optimization in Single and Multilayer In Vitro Cell Models
Source: Cancers (Basel). 2018 Mar 20;10(3):84. doi: 10.3390/cancers10030084 (PMC5876659; doi:10.3390/cancers10030084)
Supplement: Supplementary file 1 [file cancers-10-00084-s001.pdf]

Supplementary Material

Peptide Mediated In Vivo Tumor Targeting of Nanoparticles through Optimization in Single and Multilayer In Vitro Cell Models

Celina Yang, Kyle Bromma and Devika Chithrani

1. Supplementary Section S1

1.1. Testing of Stability of GNP Constructs in Buffer Solution with UV-VIS Spectrometry

The stability of the GNP constructs is tested in buffer solutions (PBS). Unmodified (citrate-capped) GNPs aggregate in buffer solutions and the shape of UV-VIS spectrum is distorted as shown in (Figure S1B), while the shape of the modified GNP in buffer solution spectrum remain similar to the shape of the GNP constructs in water (Figure S1A).

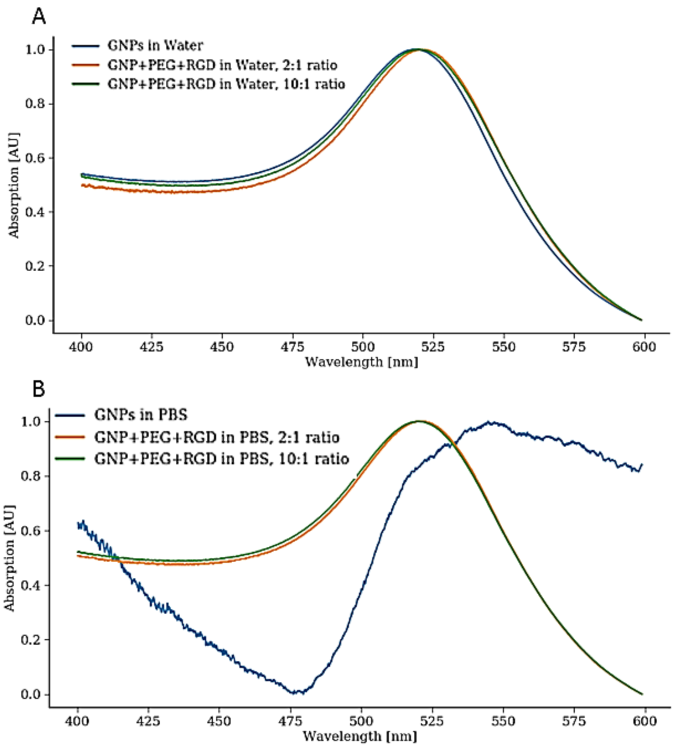

Figure S1. UV-VIS spectra of GNP constructs (**A**) in water; (**B**) in PBS buffer solution.

1.2. Testing of Stability of GNP Constructs in Buffer Solution with Dynamic Light Scattering (DLS)

The stability of the GNP constructs in buffer solutions (PBS) is also measured through dynamic light scattering (DLS). While modified GNPs in buffer solution have no significant change in size compared to modified GNPs in water (Figure S2B,C), there is a significant increase in size for the unmodified GNPs in buffer solution compared to the unmodified GNPs in water (Figure S2A).

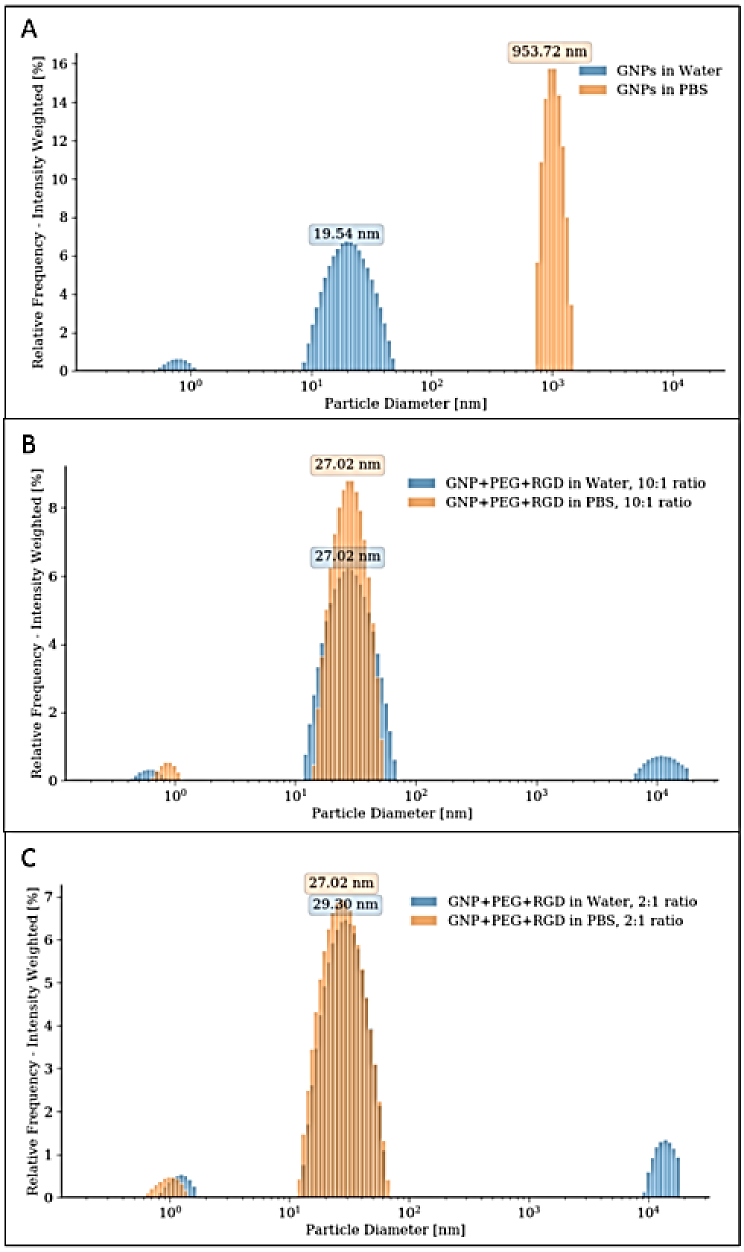

Figure S2. DLS measurements of GNP constructs.

1.3. *Summary of GNP Construct Characterization in Water and Buffer Solution*

Table S1. Summary of GNP constructs characterization.

**Sample**	**Dynamic light scattering (DLS) diameter (nm)**	**Dynamic light scattering (DLS) diameter (nm)**	**Zeta potential (mV)**	**Zeta potential (mV)**	**UV-Vis peak wavelength (nm)**	**UV-Vis peak wavelength (nm)**
Solvent	Water	PBS	Water	PBS	Water	PBS
GNP	19.5 ± 0.2	1154.0 ± 0.1	-0.3.1 ± 1.5	-34.3 ± 1.0	518.5	544.8
GNP-PEG-RGD: 2:1	28.6 ± 0.3	25.6 ± 0.2	-8.5 ± 0.5	-2.7 ± 0.9	519.9	521.3
GNP-PEG-RGD: 10:1	28.1 ± 0.2	26.6 ± 0.2	-5.8 ± 0.9	3.8 ± 0.9	519.8	520.3

2. Supplementary Section S2

2.1. *In Vivo Comprehensive Acute and Physical Toxicity Assay*

SCID mice were injected with gold nanoparticle formulations and sacrificed 24 h after injection where blood was collected from the tail vein; if this was not technically possibly the saphenous vein or direct terminal cardiac puncture was used as an appropriate substitute. Samples were then centrifuged at 14,000 rpm and serum was removed from the mixture and assessed for hepatotoxicity, nephrotoxicity, and electrolytes using an Autoanalyzer (Applied Biosystems). Mice were observed every 2 days for signs of general toxicity which include, but are not limited to: body weight changes, dull sunken eyes, interrupted breathing, and lethargy. The results of the toxicity assay are shown in the Table S2.

Table S2. *In vivo* comprehensive acute and physical toxicity assay.

	**Hepatotoxicity (U/L) (n = 4)**		
	Control	GNPs (24 h after)	Normal range
ALB	28	28	21–34
ALP	67	67	28–94
ALT	52	52	28–194
TBIL	4	5	4–5
Nephrotoxicity (mmol/L) (n=4)			
	Control	GNPs (24 h after)	Normal range
CRE	1.8	1.5	1.5–3.0
BUN	15.3	14.7	12.1–20.5
Electrolytes and Carbohydrates (mmol/L) (n = 4)			
	Control	GNPs (24 h after)	Normal range
Ca^+	1.8	1.5	1.5–3.0
PHOS	1.8	1.3	2.6–3.6
GLU	9.7	9.4	8.5–18.6
K^+	5.7	4.6	3.8–10.0
Na^+	145	147	143–150
Serum proteins (U/L) (n = 4)			
	Control	GNPs (24 h after)	Normal range
TP	37	31	30–40
GLOB	19.5	22.4	18–82
